# 2D Magnetic Manipulation of a Micro-Robot in Glycerin Using Six Pairs of Magnetic Coils

**DOI:** 10.3390/mi13122144

**Published:** 2022-12-04

**Authors:** Qigao Fan, Jiawei Lu, Jie Jia, Juntian Qu

**Affiliations:** 1 College of Internet of Things Engineering, Jiangnan University, Wuxi 214000, China; 2 Shenzhen International Graduate School, Tsinghua University, Shenzhen 518055, China; 3 Jiangsu Key Laboratory of Advanced Food Manufacturing Equipment and Technology, Wuxi 214000, China

**Keywords:** electromagnetic coil drive system, micro-robot, auto disturbance rejection technology, position servo, Kalman filter

## Abstract

This paper demonstrates the control system of a single magnetic micro-robot driven by combined coils. The combined coils consist of three pairs of Helmholtz coils and three pairs of Maxwell coils. The rotating magnetic field, gradient magnetic field, and combined magnetic field model of the combined coils were analyzed. To make the output magnetic field quickly converge to the reference point without steady-state error, the discrete-time optimal controller was designed based on the auto disturbance rejection technology. We have designed a closed-loop controller based on a position servo. The control system includes the position control and direction control of the micro-robot. To address problems with slow sampling frequency in visual feedback and inability to feed real-time position back to the control system, a Kalman filter algorithm was used to predict the position of the micro-robot in two-dimensional space. Simulations and experiments were carried out based on the proposed structure of combined coils and control scheme. The experimental results demonstrated the uniformity and excellent dynamic performance of the generated magnetic field.

## 1. Introduction

In recent years, the broad attention on micro-robots has resulted in their rapid growth. Because of their small volume and flexibility in narrow and complex environments [1,2], unrestricted and controllable micro-robots are widely used in various fields. In industry, they can be used for micro-manipulation, transportation, classification, and micro-assembly [3] of micro-objects. The high liquidity and repeatability of micro-robots make them effective tools for interacting with biological cells, which can be combined with cell operations. For example, micro-robots can be operated at the cellular or subcellular level, allowing effective in vitro interactions for moving and classifying cells [4,5,6]. The integration of microfluidic chips based on micro-electromechanical system (MEMS) with micro-robot technology is an innovation in biomedicine [7,8,9]. High-precision motion control and high-propulsion pumping machines enable micro-robots to carry multiple objects and transport them to a desired location in microfluidic chips [10]. Applications of micro-robots in vivo are broad and include minimally invasive surgery, targeted drug delivery, brachytherapy, hyperthermia, the removal of obstacles by mechanical means, and serving as fundamental structures in the body. Micro-robots can be manipulated remotely in the human body at the micro/nanoscale, including the circulatory system, urinary system, and central nervous system, facilitating applications of capsule endoscopy [11,12], ophthalmic surgery [13], and cardiovascular interventional surgery [14,15]. Therefore, the development of micro-robots will undoubtedly contribute to multi-field developments.

Micro-robots are small and cannot be powered by a built-in power supply, so they can only be driven externally. A number of driving methods have been developed, such as the use of dielectrophoretic force generated by electric fields to drive micro-scale dielectric particles [16], piezoelectric driving [17], thermal driving [18], electro-osmotic force driven [19] by biological bacteria [20,21], and micro-motors [22] driven by chemical fuel. All of these driving methods have certain limitations, especially when applied to biomedicine. Specifically, piezoelectric driving requires high voltage, the thermal driving method can easily result in excess temperatures, and the biological bacterial driving method requires monitoring to maintain low cytotoxicity. External magnetic fields [23] are also viable power sources for driving micro-robots. Magnetic fields have proven to be harmless to organisms [23,24] and have the ability to penetrate deep tissues. Therefore, the magnetic force and moment generated by magnetic fields can be applied to micro-robots without being affected by biological fluids [25].

There are usually two methods to realize magnetic field driving of micro-robots. The first method is generating the required magnetic field with a permanent magnet. For instance, the in vivo capsule endoscope can be driven with an in vitro handheld permanent magnet [11]. Nguyen and others developed the Stereotaxis Niobe system (Niobe^®^, Stereotaxis, Inc., St. Louis, MO, USA), which adopts the magnetic field driving method to drive a permanent magnet for catheter operation [26]. However, the permanent magnet driving method normally requires an additional manipulator to control the movement and rotation of the permanent magnet; compared with the second driving method based on electromagnetic coils, this method cannot generate any real-time controlled magnetic field. Therefore, many studies have selected magnetic systems driven by electromagnetic coils [27,28,29,30,31,32,33,34,35]. In [29], an enhanced electromagnetic driving device with a large workspace was developed, which could realize the trajectory tracking of magnetic particles with a proportion integration differentiation (PID) controller in two-dimensional and three-dimensional space. In [36], a position estimator was designed according to the dynamic model of the micro-robot, and trajectory tracking in the two-dimensional space of the micro-robot was realized with a self-made electromagnetic operation system.

However, the two electromagnetic driving devices mentioned above cannot produce constant magnetic flux density and magnetic field gradient. They can only be estimated approximately when analyzing the magnetic force model such that the precise dynamic model of the micro-robot cannot be obtained, which poses a higher challenge to the precise motion control of the micro-robot. As Helmholtz coils can produce a magnetic field with constant flux density, when energizing a sinusoidal current, they can produce a rotating magnetic field, whereas Maxwell coils can produce a constant magnetic field gradient. In context, micro-robots can therefore be rotated by the rotating magnetic field of a Helmholtz coil, and driven by the magnetic field gradient generated by a Maxwell coil. In [37], an intravascular electromagnetic drive system was proposed that consisted of three pairs of Helmholtz coils and one pair of Maxwell coils. The rotating magnetic field generated by the Helmholtz coils was used for the steering and rotating motion of the micro-robot, and the gradient magnetic field generated by the Maxwell coils was used for the forward motion of the micro-robot. The feasibility of the scheme was subsequently verified via experiment. In our study, three pairs of Helmholtz coils and three pairs of Maxwell coils were used to generate uniform magnetic field and uniform gradient magnetic field in space, respectively.

Some researchers have studied the motion control methods of micro-robots. In one such study, Helmholtz electromagnetic coils were manufactured to guide the micro-robot to a desired position [38]. Based on analysis of the dynamic model of the micro-robot, a controller based on neuro-fuzzy network was designed. Finally, the performance of the control system was verified by experiments. An optimal path planner based on particle swarm optimization algorithm and a robust model predictive trajectory tracking controller were designed. Fluorescent imaging was used for visual feedback, and the automatic control of the magnetic micro-robot was realized [39]. To prove the controllability and observability of the system, a micro-robot motion control method combining high-gain observer and adaptive backstepping control was proposed in [40]. While simulations and experiments verify the effectiveness and robustness of this method, some important issues have been ignored in these studies. To achieve high-precision driving in the micro-robot, it is necessary to accurately control the input current of the coil [41]. However, the inductance of the coils causes a large time delay in the system, such that when it is necessary to generate a high-frequency magnetic field, the tracking performance of the micro-robot will become unsatisfactory. As the working time increases, the coil temperature rises, and the linear relationship between the input current and the output magnetic field is destroyed, causing some interference in the system. Another problem worth studying is that industrial cameras are widely used to directly obtain the position information of micro-robots. Due to the influence of the sampling frequency in the visual feedback, the real-time position of a micro-robot cannot be immediately fed back to the control system, so the precise path tracking of the micro-robot cannot be realized.

In this paper, we propose a full-state feedback controller based on auto disturbance rejection technology to solve the asynchronous problem between the magnetic field generated by the combined coils and the input current. A circuit board based on a power inverter was used to control the voltage applied to the coil, and simulation analysis was performed in MATLAB software. At the same time, the step response, sinusoidal response, and impulse response were simulated for separate tracking. Compared with traditional PID control and open-loop control, the designed controller achieved two important functionalities: (1) the output magnetic field and magnetic field gradient could converge to the reference point; (2) optimal transient response was achieved. The control system includes a direction control module and a position control module. The direction control module was configured as a closed loop of the direction variable, and the position control module was configured as three closed loops of position, velocity, and acceleration variables, respectively. As the sampling frequency in visual feedback was slow and the real-time position could not be fed back to the control system immediately, a Kalman filter algorithm was used to predict the position of the micro-robot.

The rest of the paper is organized as follows. In Section 2, we introduce the structure of the electromagnetic coil and the dynamic model of the electromagnetic coil and the micro-robot. In Section 3, we describe the designs of the driving circuit and auto disturbance rejection controller as well as the driving model of the electromagnetic coils. In Section 4, we propose the dual closed-loop control, with which a Kalman filter algorithm is used for position prediction. Section 5 presents the simulation results of the Maxwell and Helmholtz coils, illustrates the motion control experiments for driving the micro-robot along a square and S-shape and plots for the trajectory tracking error, and provides an evaluation of the performance of the Kalman filter. The experimental results demonstrate that the proposed Kalman filter algorithm can predict the position of the control scheme effectively and improve the dynamic performance of the system. Finally, the conclusion of this work is given in Section 6.

## 2. Electromagnetic Coil Structure and Model

### 2.1. Electromagnetic Coil Structure

**Figure 1:**
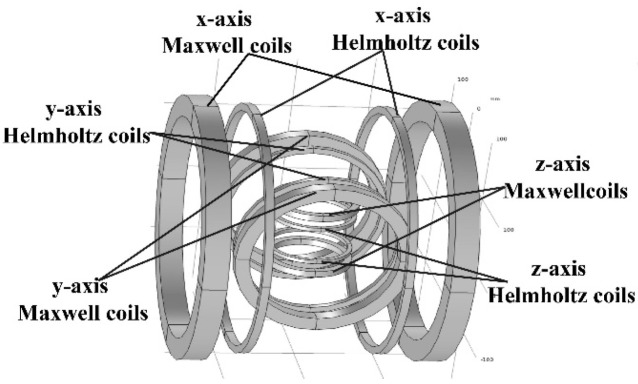
Combined coil structure.

**Figure 2:**
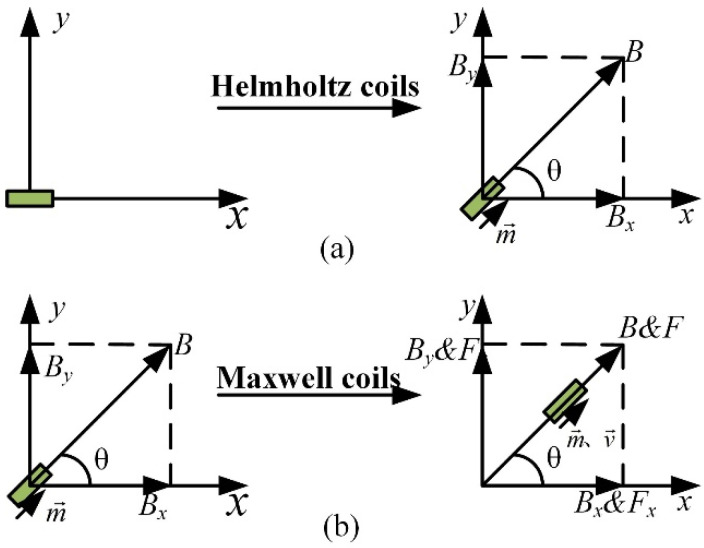
(**a**) Helmholtz coil driving principles. (**b**) Maxwell coil driving principles.

The coil structure that was used in the magnetic driving system is shown in Figure 1. The combined coils included three pairs of Helmholtz coils and three pairs of Maxwell coils, which were mutually perpendicular along the *x*-axis, *y*-axis, and *z*-axis. The distance between the Helmholtz coils was equal to the radius of the coils. Currents of equal value and direction that were injected into the two coils could produce constant magnetic flux density in the central area. If the currents of the two coils were in opposite directions, a rotating magnetic field was generated that could align the micro-robot in a desired direction, as shown in Figure 2a. The distance between the Maxwell coils was $\sqrt{3}$ times of the radius, and the two coils were supplied with currents of equal value and opposite directions to generate a constant magnetic field gradient, which was employed to drive the micro-robot, as shown in Figure 2b. Therefore, a uniform magnetic torque and magnetic force could be generated in the central area of the combined coils by combining Helmholtz coils and Maxwell coils [42,43].

### 2.2. Electromagnetic Coil Model

The micro-robot could be controlled by magnetic torque and magnetic force in the magnetic field. Torque and magnetic force are closely related to the magnetic flux density and magnetic field gradient. The force and the magnetic torque can be expressed as follows [44,45]:

(1) $$F=V\left(M\cdot \nabla \right)B$$

(2) T=VM×B

where ***F*** is the magnetic force, ***T*** is the magnetic torque, *V* is the volume of the micro-robot, ***B*** is the magnetic flux density (T), and ***M*** is the magnetization of the micro-robot (A/m). The torque of the micro-robot is determined by the magnetic field generated by the Helmholtz coil, and the magnetic force is determined by the gradient magnetic field generated by the Maxwell coil. Therefore, it is necessary to analyze these two kinds of magnetic fields to control the steering and position of the micro-robot.

In addition, the Helmholtz coil magnetic field can produce a rotating magnetic field. The normal vector of the rotating magnetic field is defined as ***n*** = (*n_x_*, *n_y_*, *n_z_*)^T^, and the magnetic field can be expressed as

(3) $$B_{H}=\left[\begin{array}{c} B_{HX}\\ B_{HY}\\ B_{HZ} \end{array}\right]=\frac{B_{0}\cos(2\pi ft)}{\left\|\left.u\right\|\right.}\left[\begin{array}{c} n_{{}^{y}}\\ -n_{x}\\ 0 \end{array}\right]+\frac{B_{0}\sin(2\pi ft)}{\left\|\left.v\right\|\right.}\left[\begin{array}{c} n_{x}n_{z}\\ n_{y}n_{z}\\ -n_{x}{}^{2}-n_{y}{}^{2} \end{array}\right]$$

where ***B****_HX_*, ***B**_HY_*, and ***B**_HZ_* are respectively expressed as the magnetic flux density generated by the *x*-axis, *y*-axis, and *z*-axis Helmholtz coils. *f* is the frequency of the rotating magnetic field, *B_0_* is the modulus of the magnetic flux density,$\left\|\left.u\right\|\right.=\sqrt{n_{y}{}^{2}+n_{x}{}^{2}}$, $\left\|\left.v\right\|\right.=\sqrt{{\left(n_{x}n_{z}\right)}^{2}+{\left(n_{y}n_{z}\right)}^{2}+{\left(n_{x}{}^{2}+n_{y}{}^{2}\right)}^{2}}$ [46,47]. The magnetic flux density *B_0_* produced by the uniaxial Helmholtz coil in a uniform region is proportional to the input current [48]:

(4) $$B_{0}={\left(\frac{4}{5}\right)}^{3/2}\frac{\mu_{0}N}{a^{2}}I_{H}$$

where *N* and *a* respectively represent the number of turns per coil and the radius of the Helmholtz coils, $\mu_{0}$ is the vacuum permeability, and *I_H_* is the current energized by the Helmholtz coils.

When *n_x_* = *n_y_* = 0, the micro-robot will rotate in two-dimensional space, meeting the research requirements of this paper. In this scenario, only the *x*-axis and *y*-axis Helmholtz coils are generating magnetic fields:

(5) $$B_{H}=\left[\begin{array}{c} B_{HX}\\ B_{HY}\\ B_{HZ} \end{array}\right]=\left[\begin{array}{c} B_{0}\cos(2\pi ft)\\ B_{0}\sin(2\pi ft)\\ 0 \end{array}\right]$$

The magnetic flux density generated by the *x*-axis Maxwell coil in the region of the central uniform gradient magnetic field is [49]

(6) $$B_{MX}=k\left[\begin{array}{c} I_{MX}\\ -0.5I_{MY}\\ -0.5I_{MZ} \end{array}\right]\left[\begin{array}{ccc} x & y & z \end{array}\right]$$

where $k=\frac{16}{3}{\left(\frac{3}{7}\right)}^{5/2}\frac{\mu_{0}N}{a^{2}}$, *N*, and *a* represent the number of turns per coil and the radius of the coil, $\mu_{0}$ is the vacuum permeability, and *I_MX_*, *I_MY_*, and *I_MZ_* are respectively expressed as the currents injected by the *x*-axis, *y*-axis, and *z*-axis Maxwell coils.

Considering the magnetic field in other directions, the synthetic magnetic field of the Maxwell coil is

(7) $$B_{M}=B_{MX}+B_{MY}+B_{MZ}=\left[\begin{array}{ccc} 1 & -0.5 & -0.5\\ -0.5 & 1 & -0.5\\ -0.5 & -0.5 & 1 \end{array}\right]\left[\begin{array}{c} k_{MX}I_{MX}\\ k_{MY}I_{MY}\\ k_{MZ}I_{MZ} \end{array}\right]\left[\begin{array}{c} x\\ y\\ z \end{array}\right]$$

### 2.3. Dynamic Model of the Micro-Robot

It can be seen from Equation (5) that the micro-robot can be rotated in any direction in two-dimensional space by injecting dynamic current into the Helmholtz coil. However, to realize the movement of the micro-robot in the plane, the gradient magnetic field drive is required. The Maxwell coils can provide magnetic force to drive the micro-robot, and when the magnetization direction of the micro-robot is consistent with the magnetic field gradient, it has the maximum magnetic force. Therefore, in our study, the uniform magnetic field was used to change the direction of the magnetic torque of the micro-robot and the direction of the desired magnetic force.

The micro-robot, when in glycerol solution, will be affected by magnetic force, gravity, viscous resistance, friction and other forces. For a micro-robot moving in two-dimensional space, the force in the vertical direction does not need to be considered. Since a cylindrical micro-robot was used, the contact area between its surface and the bottom of the container was very small. The friction in this case is close to zero but cannot be ignored, so it was regarded as a disturbance in the system. Therefore, the dynamic equation for the magnetic micro-robot that we used is

(8) $$F_{m}+F_{d}+F_{f}=m\;\ddot{p}.$$

Here, ***F****_m_* is the magnetic force applied to the micro-robot by the external magnetic field generated by the Maxwell coil. ***F****_d_* represents the resistance of the micro-robot moving in glycerol solution. ***F****_f_* is the friction between the micro-robot and the bottom of the container. The moving medium of the micro-robot in the experiment was glycerol, which is a solution of low Reynolds number. The viscous resistance of a micro-robot placed in a low-Reynolds-number solution is [50]

(9) $$F_{d}=6\pi \eta Rv,$$

where *η* represents the dynamic viscosity of the glycerol solution, *R* is the radius of the micro-robot, and ***v*** represents the speed of the micro-robot.

It is assumed that the coil produces a magnetic field ***B****_k_(**p**)* at point ***p*** such that for a current injected into the coil *i_k_*, the *k_th_* coil produces a magnetic field ${\bar{B}}_{k}(p)$ at point ***p*** when a current of 1A is passed. Therefore, the magnetic field generated by the combined coils at point ***p*** can be expressed by the following formula:

(10) $$B(p)=\sum_{k=1}^{6}B_{k}(p)=\sum_{k=1}^{6}{\bar{B}}_{k}(p)i_{k}=[B_{1}(p)\cdots B_{6}(p)]\left[\begin{array}{c} i_{1}\\ \vdots \\ i_{6} \end{array}\right]=\beta (p)I$$

where *k* = 1, 2,…, 6 respectively represent the *x*-axis, *y*-axis and *z*-axis of Helmholtz coils and the *x*-axis, *y*-axis, and *z*-axis of Maxwell coils. ***I*** denotes ${\left[i_{1}\cdot \cdot \cdot i_{6}\right]}^{T}$. The gradient components of the generated magnetic field are

(11) $$\left\{\begin{array}{c} \frac{\partial B(p)}{\partial x}=\left[\begin{array}{ccc} \frac{\partial {\bar{B}}_{1}(p)}{\partial x} & \cdots & \frac{\partial {\bar{B}}_{6}(p)}{\partial x} \end{array}\right]\left[\begin{array}{c} i_{1}\\ \vdots \\ i_{6} \end{array}\right]=\beta_{x}(p)I\\ \frac{\partial B(p)}{\partial y}=\left[\begin{array}{ccc} \frac{\partial {\bar{B}}_{1}(p)}{\partial y} & \cdots & \frac{\partial {\bar{B}}_{6}(p)}{\partial y} \end{array}\right]\left[\begin{array}{c} i_{1}\\ \vdots \\ i_{6} \end{array}\right]=\beta_{y}(p)I\\ \frac{\partial B(p)}{\partial z}=\left[\begin{array}{ccc} \frac{\partial {\bar{B}}_{1}(p)}{\partial z} & \cdots & \frac{\partial {\bar{B}}_{6}(p)}{\partial z} \end{array}\right]\left[\begin{array}{c} i_{1}\\ \vdots \\ i_{6} \end{array}\right]=\beta_{z}(p)I \end{array}\right.$$

As the direction of the internal magnetic torque of the magnetic micro-robot will be consistent with the direction of the external magnetic field, and as the magnetic field generated by the Maxwell coil in the central area can be safely ignored, according to (1) and (2), the magnetic torque and magnetic force of the micro-robot in the magnetic field can be calculated as

(12) $$\left[\begin{array}{c} B\\ F \end{array}\right]=\left[\begin{array}{c} \beta (p)\\ m^{T}\beta_{X}(p)\\ m^{T}\beta_{Y}(p)\\ m^{T}\beta_{Z}(p) \end{array}\right]\left[\begin{array}{c} i_{1}\\ \vdots \\ i_{6} \end{array}\right]=\Lambda_{B,F}(m,\;p)I$$

Given the desired path of the micro-robot, the required magnetic field ***B****_des_* and magnetic force ***F****_des_* can be determined according to the desired motion direction and position. The input current of each coil can be obtained by solving Equation (12):

(13) $$I=\Lambda^{-1}{}_{B,F}(m,p)\left[\begin{array}{c} B_{des}\\ F_{des} \end{array}\right]$$

## 3. Optimal Control of Magnetic Field Drive

### 3.1. Design of Driving Circuit

**Figure 3:**
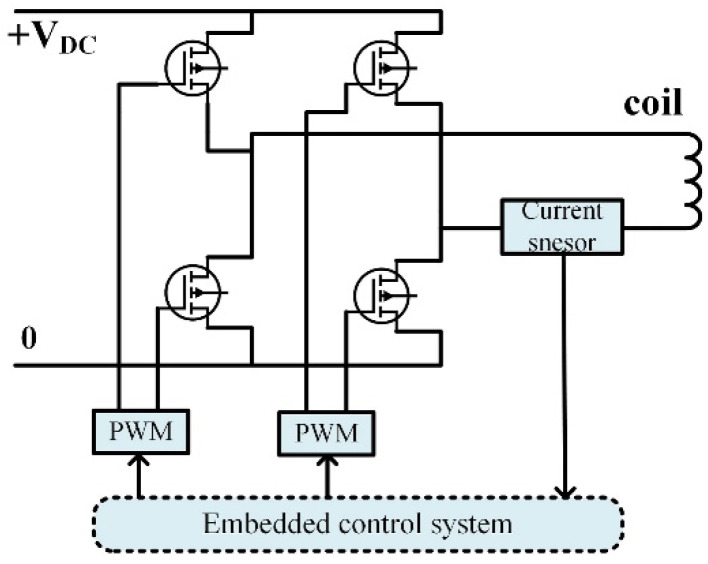
Schematic diagram of coils drive board.

The coil drive module was designed based on the power inverter. The control program is executed by the microprocessor, at which point the control input calculated in each loop is converted by the DSP into a pulse width modulation (PWM) signal through the power inverter (switching frequency is 20 kHz) on a full bridge circuit control board as shown in Figure 3.

### 3.2. Design of Auto Disturbance Rejection Controller

**Figure 4:**
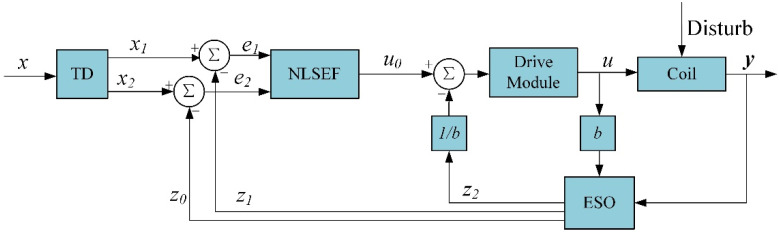
Block diagram of auto disturbance rejection controller.

The design of this component is based on auto disturbance rejection control technology to solve the time delay problem caused by the self-induced coil currents and realize the rapid response of the coil-generated magnetic fields to the input current. Figure 4 shows the structure diagram of the auto disturbance rejection controller, which is composed of a tracking differentiator (TD), an extended state observer (ESO), and a state error feedback control law (NLSEF).

For current control, the discrete equation for the transition process of the tracking differentiator is expressed as

(14) $$\left\{\begin{array}{l} x_{1}(k+1)=x_{1}(k)+Tx_{2}(k)\\ x_{2}(k+1)=x_{2}(k)+Tfst[x_{1}(k)-x(k),x_{2}(k),r,h] \end{array}\right.$$

In Formula (14), *x* represents the input current as the input signal of the auto disturbance rejection controller, *x*_1_(*k*) is the tracking of the input signal *x*(*k*), *x*_2_(*k*) is the differential of *x*_1_(*k*), and *T* is the period of discrete control. *r* and h are adjustable parameters in the tracking differentiator TD. The larger that *r* is, the faster the tracking speed becomes, but this may exacerbate overshooting. *h* is the filter parameter: the noise filtering effect improves with the increase of *h*. Thus, we adjusted the parameters through the output waveform of the tracking differentiator. The optimal control function of the system is

(15) $$fst\left[x_{1}\left(k\right)-x\left(k\right),x_{2}\left(k\right),r,h\right]=-\left\{\begin{array}{c} ra/d,\left|a\right|\leq d,d=rh\\ r\mathrm{sgn}\left(a\right),\left|a\right|>d,d=rh \end{array}\right.$$

(16) $$a=\left\{\begin{array}{c} x_{2}\left(k\right)+\frac{a_{0}-d}{2}\mathrm{sgn}\left[y\left(k\right)\right],\left|y\left(k\right)\right|>d_{0},d_{0}=dh\\ x_{2}\left(k\right)+\frac{y\left(k\right)}{h},\left|y\left(k\right)\right|\leq d_{0},d_{0}=dh \end{array}\right.$$

(17) $$y\left(k\right)=x_{1}\left(k\right)-x\left(k\right)+hx_{2}\left(k\right)$$

(18) $$a_{0}=\sqrt{d^{2}+8r|y(k)|}$$

The second-order extended state observer is

(19) $$\left\{\begin{array}{l} e(k)=z_{1}(k)-y(k)\\ z_{1}(k+1)=z_{1}(k)+T[z_{2}(k)-\beta_{01}fal(e(k),\alpha_{1},\delta_{1})+bu(k)]\\ z_{2}(k+1)=z_{2}(k)-T\beta_{02}fal(e(k),\alpha_{1},\delta_{1}) \end{array}\right.$$

In the above Formula (19), *y*(*k*) is the output current based on the active disturbance rejection controller. The estimation of output current is represented by *z*_1_(*k*), and *z*_2_(*k*) estimates the total disturbance of the system. *α*_1_, *δ*_1_, *β*_01_, and *β*_02_ are the four adjustable parameters of the expanded state observer; we usually set *α*_1_∈(0,1). To make the system disturbance observable by the extended state observer in time, the *fal* function is added to the extended state observer to achieve rapid convergence. Note that it is necessary to make *α*_1_ as small as possible to achieve faster tracking. *δ*_1_ is the filtering parameter of the ESO; in our case, usually set as *δ*_1_ = 0.01. The dynamic performance of the system is largely affected by *β*_01_ and *β*_02_. The estimation of the system state variables is mainly controlled by *β*_01_, while the estimation of the system disturbance is controlled by *β*_02_. Larger *β*_01_ and *β*_02_ are required to obtain a faster estimation convergence rate, but overlarge *β*_01_ and *β*_02_ will generate high-frequency noise and produce oscillation divergence. Usually, we set *β*_01_ = 1/T and *β*_02_ = 1/(5T^2^). The state variables of the system can be accurately observed by *z*_1_(*k*), and the disturbance of the system can also be accurately estimated by *z*_2_(*k*) after the parameters are set completely. The nonlinear combination function *fal* is

(20) $$fal(e(k),\alpha_{1},\delta_{1})=\left\{\begin{array}{c} |e(k){|}^{\alpha_{1}}\mathrm{sgn}(e(k)),|e(k)|>\delta_{1}\\ e(k)\delta_{1}{}^{\alpha_{1}-1},|e(k)|\leq \delta 1 \end{array}\right.$$

According to the output of the tracking differentiator and the ESO, the first-order nonlinear state feedback control law can be constructed as follows:

(21) $$\left\{\begin{array}{l} e_{1}(k)=x_{1}(k)-z_{1}(k)\\ u_{0}(k)=\beta_{1}fal(e_{1}(k),\alpha_{01},\delta_{01})\\ u(k)=u_{0}(k)-z_{2}(k)/b \end{array}\right.$$

where *u*(*k*) is the output control quantity of the auto disturbance rejection controller and *α_0_*_1_, *δ*_01_, and *β*_1_ are the three adjustable parameters in the nonlinear feedback control law. Generally, *α*_01_∈(0,1) and *δ*_01_ = 0.01; their actual definition is similar to the parameters for *α*_1_ and *δ*_1_ in the ESO. The response speed of the system can be enhanced by increasing *β*_1_, but it will cause oscillation, so it is necessary to adjust *β*_1_ according to the overall control performance of the system.

## 4. Closed-Loop Control System

**Figure 5:**
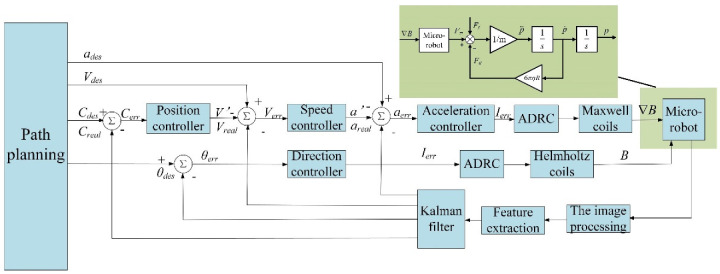
Schematic diagram of micro-robot planar path-tracking control.

In our study, we generally adopted a dual closed-loop control structure. The specific control system diagram is shown in Figure 5.

In Figure 5, *θ_des_* and *C_des_* are the expected movement direction and position of the micro-robot on the basis of the planned path. The Kalman filter algorithm is used to predict the motion states of the micro-robot to address real-time delay in feeding back position due to the slow sampling frequency of visual feedback. The difference between the filtered real-time feedback motion direction *θ_real_* and the feedback real-time position *C_real_* is calculated to acquire the momentary motion direction error *θ_err_* and position error *C_real_*. The real-time output magnetic field and magnetic field gradient are applied to the micro-robot via direction and position control, respectively. The control system employs a dual closed-loop structure, which is composed of a position tracking loop with the real-time position and a direction control loop with the movement direction. The micro-robot position closed loop is composed of a position controller, a speed controller, and an acceleration controller. The output of the previous controller is the input of the latter. The final output current is injected into the Helmholtz coil and Maxwell coil through the designed ADRC to quickly generate the magnetic field for steering and movement, driving the micro-robot to follow the planned path. The dual closed-loop structure was adopted for the control system because, in the high-precision real-time control process of the micro-robot, the position error and the direction error cannot be completely eliminated at the same time in most cases. In addition, the position controller, speed controller, and acceleration controller all adopt traditional PID control. In our study, the working environment of the micro-robot was a glycerin solution with low Reynolds number; the inertia of the micro-robot when moving in it could basically be ignored, so there was no need to use the differential link to realize advance correction. Therefore, the direction controller adopted PI control.

**Figure 6:**
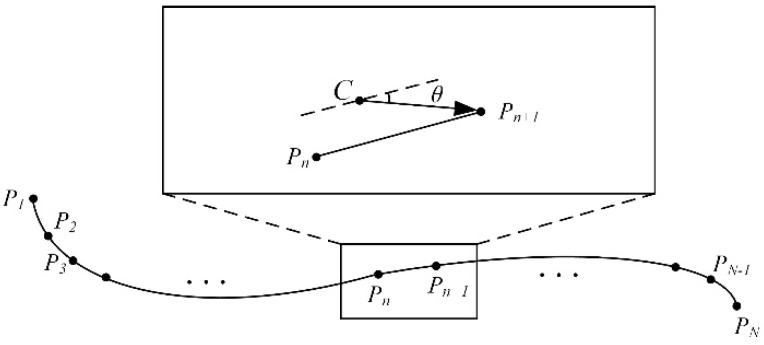
Schematic diagram of micro-robot path tracking.

Figure 6 is a schematic diagram of the path tracking of the micro-robot. Assuming that the position of the micro-robot is not on the planned path *P_n_P_n_*_+1_, it is located at *C*, but the movement direction is consistent with the expected path direction. Moving in the direction of the path, the direction error *θ_err_* will inevitably increase. Similarly, when the micro-robot is in the desired position, the direction of movement is not consistent with the desired direction, such that the position error *C_real_* will inevitably increase. Consequently, use of a dual closed-loop structure can achieve high-precision path tracking.

### Location Prediction

In the above-mentioned closed-loop control process, the position of the micro-robot is fed back using vision-based method during the movement. The actual position is compared with the expected position to obtain the next control signal. Therefore, the accuracy of the feedback position is directly related to the precise movement of the micro-robot. In actual visual feedback, since the frequency of the visual feedback is less than the control frequency, acqusition of position data at the control moment is unreliable between the two picture frames. We assumed a *k*th control signal in the two adjacent frames of *i* − 1 and *i* such that, in the actual movement process, the micro-robot will have passed the moment in the *i* − 1 picture to the *k*th control signal. At this moment, data that can be visually fed back are still the data in the *i* − 1th picture, such that the position information feedback of the micro-robot is not accurate. Therefore, predicting the position of the micro-robot at the time of the control signal between the *i* − 1 and *i* frame pictures is highly necessary to improve control accuracy.

To address this problem, we employed a Kalman filter to predict the position of the micro-robot between the two picture frames, which mainly include the prediction stage and the update stage. In the prediction stage, the momentary state is estimated by the state of the micro-robot at the previous moment, and then the state information of the micro-robot is forwarded to the update stage for updating the predicted state information to obtain more accurate position information. The Kalman filter does not predict all information at each control time; rather, the feedback information is used when the control time overlaps with the image feedback time.

Details of the adopted Kalman filter algorithm and the derived state transition matrix of the micro-robot are provided in the S1 section of the supplementary material.

## 5. Simulation and Experiments

### 5.1. Simulation

#### 5.1.1. Magnetic Field of Combined Coils

To improve the accuracy of rotation and movement of the micro-robot, the uniformity of the magnetic field generated by the electromagnetic coils is very important. In this section, we discuss our finite-element analysis of the constant magnetic field, gradient magnetic field, and rotating magnetic field generated by the combined coil. The analysis was conducted using the multi-physics simulation software COMSOL according to the structure model of the combined coils.

The specific parameters of the combined coils are shown in Table 1.

In Table 1, HX represents the *x*-axis Helmholtz coil, and MX represents the *x*-axis Maxwell coils.

**Figure 7:**
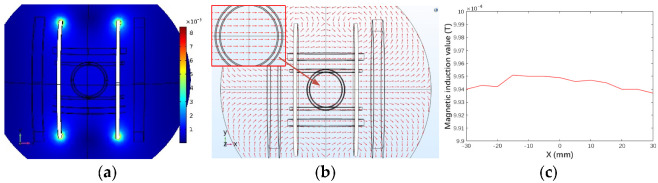
*x*-axis Helmholtz coil simulation. (**a**) The modulus of magnetic flux density. (**b**) The vector of magnetic flux density. (**c**) Magnetic induction value distribution in the working space.

**Figure 8:**
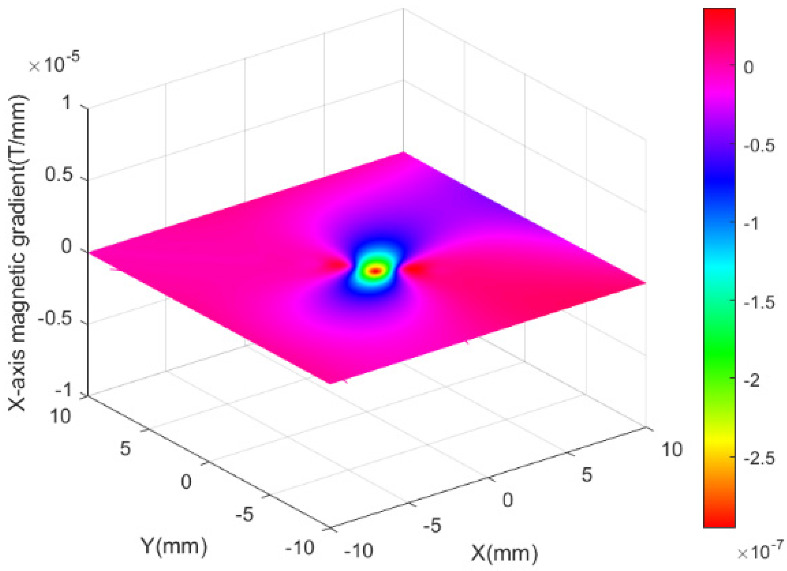
Magnetic field gradient of *x*-axis Helmholtz coil.

Figure 7 shows the simulation results of the magnetic field generated by the *x*-axis Helmholtz coil in the plane when a 4 A current was applied. Figure 7a shows the modulus field diagram of the magnetic flux density. To show the direction and intensity of the magnetic flux density, the vector field diagram of the magnetic flux density was simulated, as shown in Figure 7b. As shown in Figure 7c, a constant magnetic flux density of approximately 1 mT could be generated in the center of the workspace. Figure 8 shows the gradient distribution of the magnetic field generated by the *x*-axis Helmholtz coil on the central plane of space. Figure 8 shows that the magnetic field gradient is approximately 0. The simulation results demonstrate that Helmholtz coils can produce uniform magnetic flux density on the central plane. Therefore, Helmholtz coils could regulate the direction of the micro-robot by rotating it, but it could not apply propulsive force.

**Figure 9:**
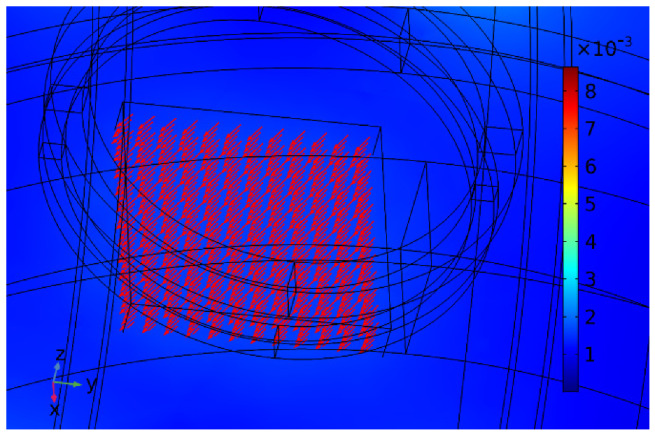
Three-axis Helmholtz coils magnetic flux density distribution.

Given that Helmholtz coils can produce a uniform magnetic field, we injected a constant 4A current into three pairs of Helmholtz coils to analyze the resulting magnetic field. As shown in Figure 9, the superposed magnetic field generated by three pairs of coils in the central area is also uniform.

To verify the effectiveness of the rotating magnetic field, the *z*-axis is selected as the rotation axis, and the magnetic flux density is obtained by Equation (5):

(22) $$B_{H}=\left[\begin{array}{c} B_{HX}\\ B_{HY}\\ B_{HZ} \end{array}\right]=\left[\begin{array}{c} B_{0}\cos(2\pi ft)\\ B_{0}\sin(2\pi ft)\\ 0 \end{array}\right]$$

From the simulation results of the uniaxial coil, the linear relationship between the magnetic flux density generated by each pair of Helmholtz coils and the input current can be approximated as follows:

(23) $$\left\{\begin{array}{c} B_{HX}=0.25I_{HX}\\ B_{HY}=0.25I_{HY}\\ B_{HZ}=0.25I_{HZ} \end{array}\right.$$

Combining (22) and (23) and setting *B*_0_ = 1 mT and *f* = 1 Hz, the input current of coil can be obtained by

(24) $$\left\{\begin{array}{c} I_{HX}=4\cos(2\pi t)\\ I_{HY}=4\sin(2\pi t)\\ I_{HZ}=0 \end{array}\right.$$

The simulation results of the rotating magnetic field rotating around the *z*-axis are shown in Figure S1. Since the period of the rotating magnetic field is 1 s, we recorded the magnetic flux density distribution in the x–y plane every 0.125 s.

**Figure 10:**
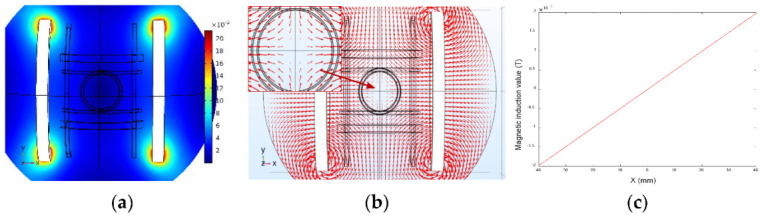
*x*-axis Maxwell coil simulation. (**a**) The modulus of magnetic flux density. (**b**) The vector of magnetic flux density. (**c**) Magnetic induction value distribution in the working space.

**Figure 11:**
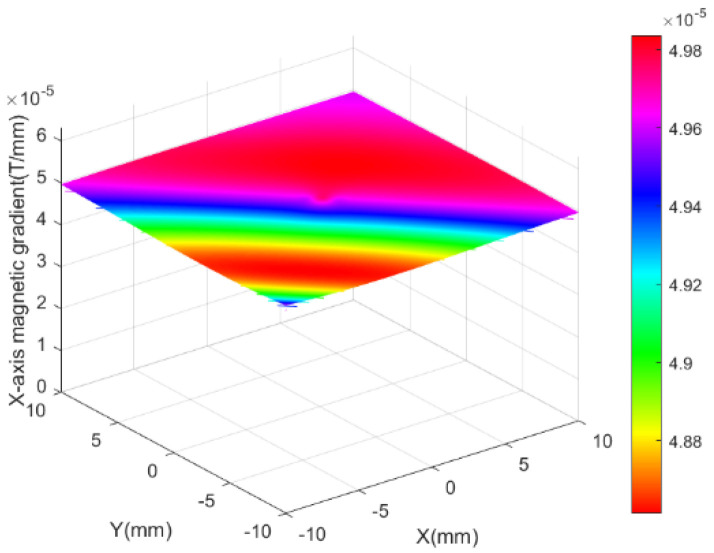
Magnetic field gradient of *x*-axis Maxwell coil.

Figure 10 and Figure 11 present the simulation results of the *x*-axis Maxwell coil, which shows that the gradient of the magnetic flux density was uniform and that a magnetic field gradient of 0.05 T/m was generated when 4A current was injected. Therefore, it was possible to obtain different magnetic forces for driving the micro-robot by changing the supply current in the Maxwell coil. Note, therefore, that reversal of the direction of the injected current between the two Maxwell coils will cause the direction of the magnetic field gradient to be reversed.

**Figure 12:**
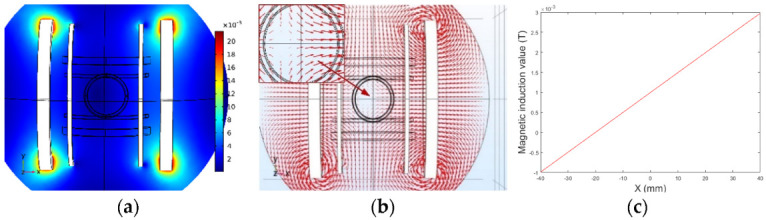
Single-axis combined coils simulation. (**a**) The modulus of magnetic flux density. (**b**) The vector of magnetic flux density. (**c**) Magnetic induction value distribution in the working space.

Figure 12 shows the magnetic flux density distributions generated by combining the *x*-axis Helmholtz coil and the *x*-axis Maxwell coil. The current for the Helmholtz coil and Maxwell coil was set at 4 A. The results show that a gradient magnetic field along the x direction could be generated.

**Figure 13:**
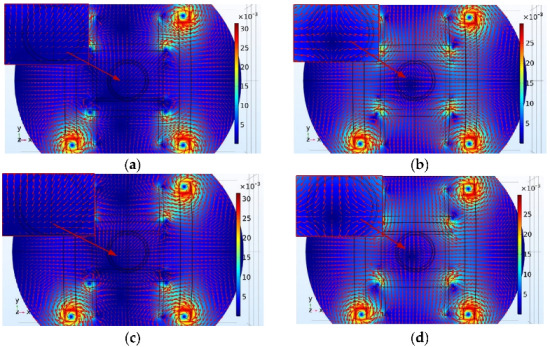
(**a**) Helmholtz *x*, *y*, *z* axes under 4 A current. Maxwell *x*, *y*, *z* axes under −4 A current. (**b**) Helmholtz *x*, *z* axes under −4 A current, Helmholtz *y*-axis under 4 A current. Maxwell *x*, *z* axes under 4 A current, Maxwell *y*-axis under −4 A current. (**c**) Helmholtz *x*, *y*, *z* axes under −4 A current, Maxwell *x*, *y*, *z* axes under 4 A current. (**d**) Helmholtz *x*, *z* axes under 4 A current, Helmholtz *y*-axis under −4 A current. Maxwell *x*, *z* axes under −4 A current, Maxwell *y*-axis under 4 A current.

To obtain the force in any direction on the plane of the micro-robot, the combined coils need to generate gradient magnetic fields in different directions. Figure 13 shows that the combined coils can generate gradient magnetic fields in different directions on the x–y plane.

#### 5.1.2. Combined Coil Drive Based on ADRC

In Section 3, we described a combined-coil current-control strategy based on ADRC to improve the fast response performance of the magnetic field. This section describes the verification of the effectiveness of this method via simulation. A simulation platform of the electromagnetic coil system was built using the MATLAB/SIMULINK environment. Since any discrete time process consists of a step response, and as any continuous signal can be approximated by a finite number of discrete steps, a discrete system model with calibrated noise was used to obtain the step response, sinusoidal response, and impulse response, respectively, to evaluate the dynamic response performance of the system. The open loop control, PI control, and auto disturbance rejection control of the combined-coils control system were respectively simulated and then compared. The control frequency of the combined coils was set to 20 kHz.

**Figure 14:**
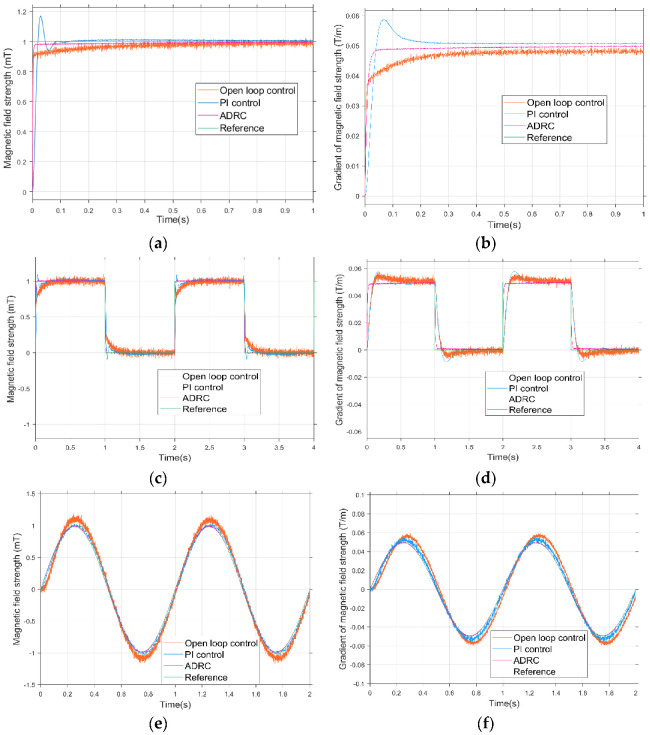
(**a**) Step-response simulation of Helmholtz coil. (**b**) Step-response simulation of Maxwell coil. (**c**) Impulse-response simulation of Helmholtz coil. (**d**) Impulse-response simulation of Maxwell coil. (**e**) Sinusoidal-response simulation of Helmholtz coil. (**f**) Sinusoidal-response simulation of Maxwell coil.

The simulation results of step response, sinusoidal response, and impulse response of the Helmholtz coils and Maxwell coils are shown in Figure 14. The Helmholtz coils and Maxwell coils were used to generate a uniform magnetic field and gradient magnetic field, so the ordinates are the magnetic flux density value and the magnetic flux density gradient, respectively.

The magnetic flux density given in Figure 14a is 1 mT, and the step value was set as t = 0 s. Upon comparing the three control methods of the *x*-axis Helmholtz coil, under open-loop control, although the coil response had no overshoot, the time to reach stability was longer than PI control and the auto disturbance rejection control methods, 400 ms in duration. When the steady state was reached, the response was slower and there was a larger steady state error. Under PI control, the coil response performance improved, but maximum overshoot was 16% and there was a steady-state error. In addition, both the PI control and open-loop control exhibited oscillations, which are harmful to the system. Finally, under the auto disturbance rejection control method proposed in this paper, the response performance was further accelerated with only 20 ms required, and there was basically no overshoot, no steady-state error, and small oscillation. The setting magnetic flux density gradient of the Maxwell coil shown in Figure 14b is 0.05 T/m. The open-loop response performance was the worst, having the largest steady state error, and the steady state time reached up to about 500 ms. PI control improved the response time to 300 ms and the maximum overshoot to 18%. Thus, the response time and steady-state performance of the coil under auto-disturbance control were greatly improved. The performance of the sinusoidal response and impulse response were also improved.

#### 5.1.3. Closed-Loop Control System

The results from our simulations show that the auto disturbance control strategy can make the magnetic field generated by the energized coil respond quickly enough to solve the time-delay problem of the combined-coil drive. This section discusses the simulation of the closed-loop control system described in Section 4 to verify the effectiveness of the proposed closed-loop control method and the effectiveness of the micro-robot path-tracking when the Kalman filter is implemented. In the simulation setting, the micro-robot tracks the step signal and the sinusoidal signal with and without the Kalman filter algorithm. The step signal and the sinusoidal signal were respectively set as

(25) $$\left\{\begin{array}{l} y_{1}=0.001m\\ y_{2}=0.001\sin\left(2\pi t\right)m \end{array}\right.$$

**Figure 15:**
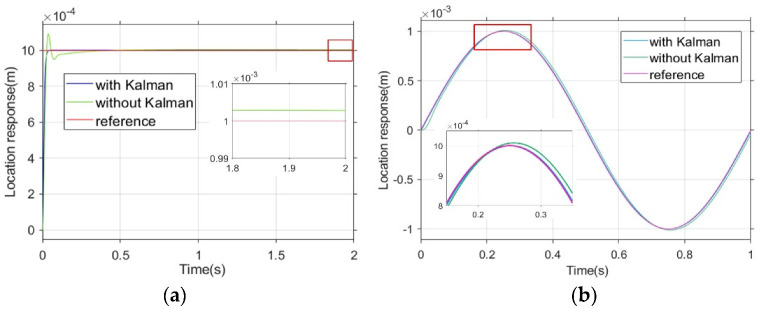
Simulation response of micro-robot tracking two signals. (**a**) Micro-robot tracking step signal. (**b**) Micro-robot tracking sinusoidal signal.

The response of the micro-robot tracking the step signal is shown in Figure 15a. According to the simulation results, the micro-robot basically has no overshoot or steady-state error under the Kalman filter algorithm while tracking the step signal. Compared with the result without Kalman filter algorithm, this approach shows better dynamic performance and steady-state performance. The response of the micro-robot tracking the sinusoidal signal is shown in Figure 15b. According to the simulation results, the micro-robot has obvious tracking error without the Kalman filter algorithm when tracking the sinusoidal signal.

### 5.2. Experiments

**Figure 16:**
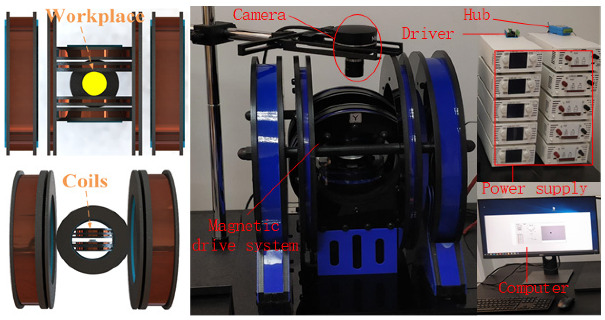
Experimental environment settings.

The micro-robot magnetic driving force generation and control system experimental platform was mainly composed of an electromagnetic coils module, a coils driving module, and a visual feedback module. The experimental environment is shown in Figure 16. The picture on the left is a partial view of the working space. The combined-coil parameters of the magnetic drive system in Figure 16 are the same as those in Table 1 for the simulation part. A cylindrical rubidium iron boron magnet with a diameter of 1 mm and a height of 1 mm was selected for the driving object. The micro-robot had a circular two-dimensional workspace with a diameter of 60 mm, and glycerin liquid with a kinematic viscosity of 350 cs was used for the moving medium of the micro-robot.

#### 5.2.1. Combined Coils Drive Based on ADRC

**Figure 17:**
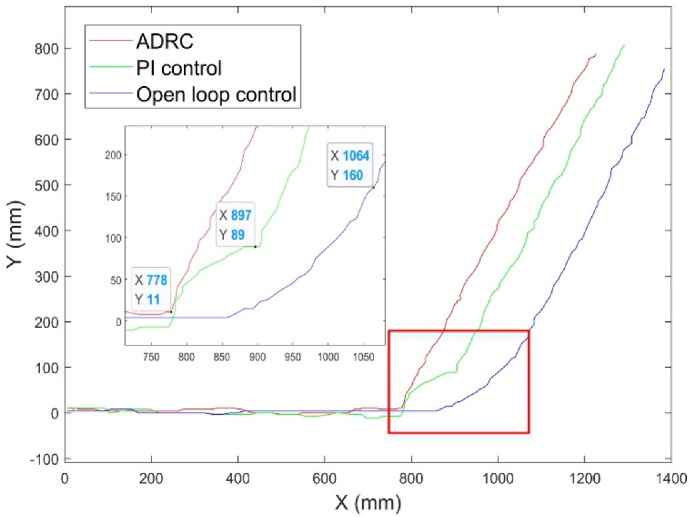
Steering tracking.

In Section 5.1.2, the effectiveness of the combined-coils current-control strategy based on ADRC was verified through simulation. In this section, we describe our experiments for the further verification of the performance of this method. The dynamic performance of the coil was determined based on its movement trajectory and time during the actual micro-robot movement. In the experiments, the initial movement direction of the micro-robot s along the *x*-axis direction. When returning the micro-robot to the origin, application of force of equivalent magnitude along both the *y*-axis and *x*-axis should result in a y = x trajectory. Similar to the simulation in Section 3, open-loop control, PI control, and the proposed auto-disturbance control methods were used to realize the movement of the micro-robot, respectively. The experimental results are shown in Figure 17.

The motion trajectory of the micro-robot under three control methods is presented in Figure 17. At the beginning of the linear motion stage, the motion of the micro-robot under the auto-disturbance control is more stable, and there is basically no time delay for the change of response. Compared with open-loop control and PI control methods, the adjustment distance of the micro-robot under auto-disturbance control method was the shortest. There was a sudden change of adjustment distance under PI control due to overshoot, and the open-loop control method generated the longest adjustment distance. Based on whole movement process observation, the micro-robot movement under auto-disturbance control was the smoothest and basically had no vibration.

#### 5.2.2. Closed-Loop Motion Control

To demonstrate the performance of the closed-loop control system, the micro-robot was driven to move along the square path and S-shape path, respectively. The performance of the Kalman filter was evaluated by observing the trajectory and error of the micro-robot.

**Figure 18:**
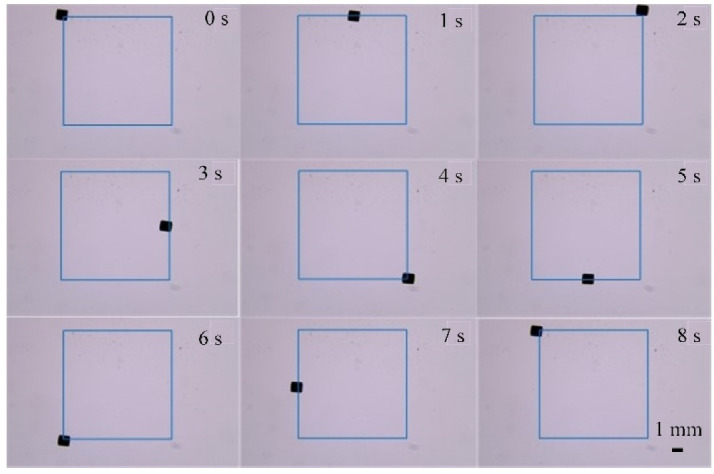
Square movement process.

First, we drove the micro-robot to move along a square path with a side length of 10 mm. Figure 18 shows the stages of the movement process of the micro-robot.

**Figure 19:**
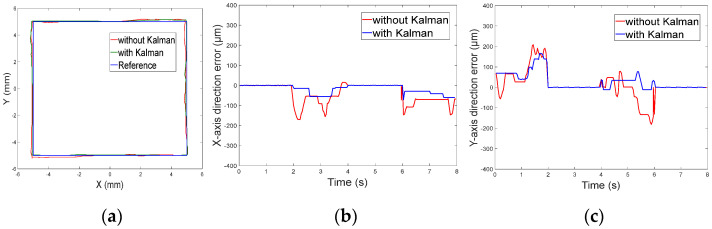
(**a**) Square trajectory. (**b**) Motion errors in *x*-axis. (**c**) Motion errors in *y*-axis.

Figure 19a shows the trajectory of the micro-robot moving along the square path. The important role that the Kalman filter played in the control system is evident in that the motion of the micro-robot was significantly smoother and the error was smaller after the Kalman filter was added as the position predictor.

Figure 19b and c respectively show the errors in the *x*-axis direction and the *y*-axis direction. It is obvious that the errors in the two directions decreased significantly under the Kalman filter. When the Kalman filter was applied in the control system, the *x*-axis direction error was reduced by about 57.3%, and the *y*-axis direction error was reduced by about 23.5%.

**Figure 20:**
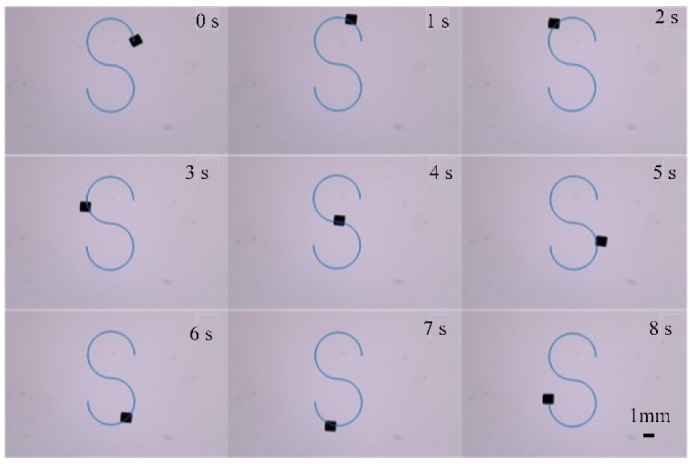
‘S’ movement process.

**Figure 21:**
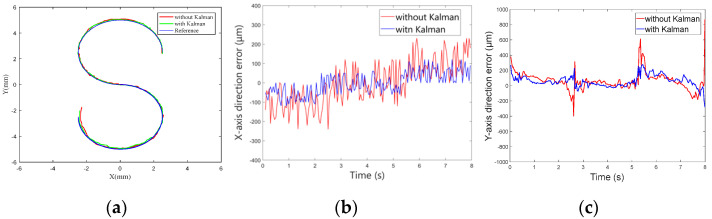
(**a**) ‘S’ motion trajectory. (**b**) Motion errors in *x*-axis. (**c**) Motion errors in *y*-axis.

Figure 20 shows the process of the micro-robot moving along the S-shape path. Figure 21a shows the trajectory of the micro-robot moving along the S-shape path. Use of the Kalman filter in the control system is apparent in these figures, as the movement of the micro-robot is more smooth and lacks excessive jitter. Using the filter, the overall dynamic performance of system was improved.

Figure 21b and c show the errors in the *x*-axis direction and the *y*-axis direction, respectively. Use of the Kalman filter in the control system resulted in the errors in the *x*-axis direction being reduced by about 53.1% and the errors in the *y*-axis direction being reduced by about 22.5%.

## 6. Conclusions

This paper demonstrates the use of combined coils to drive a magnetic micro-robot on a two-dimensional plane, for which we proposed a time-optimal control strategy based on auto disturbance rejection control technology. Compared with traditional PID controllers, the auto disturbance rejection control had better dynamic performance, and its effectiveness was verified by simulation analysis. The Kalman filter algorithm was used to predict the position of the micro-robot. Analysis of the experimental results shows that use of the Kalman filter algorithm substantially reduced the tracking errors of the micro-robot. Future studies will focus on the motion control of micro-robots in three-dimensional space and attempt experiments in more complex environments or in the context of living organisms.

## Figures and Tables

**Table 1:** Parameters of combined coils.

Parameter	HX	MX	HY	MY	HZ	MZ
resistance (Ω)	2.3	11.5	1.3	3.0	0.8	1.2
Inductance (mH)	4.397	207.2	1.514	14.82	0.1208	0.511
Coil average distance (mm)	170	294	100	173	50	87
Equivalent radius (mm)	170	170	100	100	50	50
Coil outer diameter (mm)	400	400	230	230	120	120
Coil inner diameter (mm)	248	248	151	151	66	66
Number of turns	52	449	31	156	14	39
Single copper wire diameter (mm)	1.4	1.4	1.4	1.4	1.4	1.4

## Data Availability

The datasets used or reported in the current work are available from the corresponding author on reasonable request.

## Supplementary Materials

The following supporting information can be downloaded at: www.mdpi.com/article/10.3390/mi13122144/s1.
