# Supplementary material for: 2D Magnetic Manipulation of a Micro-Robot in Glycerin Using Six Pairs of Magnetic Coils
Source: Micromachines (Basel). 2022 Dec 4;13(12):2144. doi: 10.3390/mi13122144 (PMC9784892; doi:10.3390/mi13122144)
Supplement: Supplementary file 1 [file micromachines-13-02144-s001.zip › micromachines-2016372-supplementary.pdf]

Supplementary Material

2D Magnetic Manipulation of a Micro-Robot in Glycerin Using Six Pairs of Magnetic Coils

Qigao Fan[1], *Jiawei Lu*[1], *Jie Jia*[1] *and Juntian Qu*[2,3,*]

[1.] College of Internet of Things Engineering, Jiangnan University, Wuxi 214000, China;

[2.] Shenzhen International Graduate School, Tsinghua University, Shenzhen 518055, China

[3.] Jiangsu Key Laboratory of Advanced Food Manufacturing Equipment and Technology, Wuxi 214000, China

* Correspondence: juntian.qu@sz.tsinghua.edu.cn; Tel.: +86-135-2176-6518

1. Kalman Filter Algorithm and State Transition Matrix of Micro-Robot

The Kalman filter algorithm mainly uses the state equation of the system, the measurement equation of the system, the statistical characteristics of the system measurement error, and the statistical characteristics of the white noise. In fact, the essence of the Kalman filter algorithm is its ability to estimate the minimum variance of unbiased recursion results for the linear estimation of a local state.

Suppose that the state equation and measurement equation of a discrete system are

$$X_k = \Phi_{k,k-1} X_{k-1} + \Gamma_{k,k-1} W_{k-1} \tag{S1}$$

$$Z_k = H_k X_k + V_k \tag{S2}$$

where X_k is the system state vector at time k, X_{k-1} is the system state vector at time $k-1$, $\Phi_{k,k-1}$ is the system state transition matrix, $\Gamma_{k,k-1}$ is the input matrix of the system process noise, W_{k-1} is the process noise sequence, Z_k is the system measured value at time k, H_k is the system measurement matrix, and V_k is the measurement noise sequence.

Assuming that the process noise and measurement noise in the linear discrete system of Equations (S1) and (S2) are both Gaussian white noise and there is no connection between them, the Kalman filter algorithm can be described by the following equations:

$$\widehat{X}_{k/k-1} = \Phi_{k/k-1} \widehat{X}_{k-1} \tag{S3}$$

$$P_{k/k-1} = \Phi_{k,k-1} P_{k-1} \Phi^T{}_{k,k-1} + \Gamma_{k,k-1} Q_{k-1} \Gamma^T{}_{k,k-1} \tag{S4}$$

$$K_k = P_{k/k-1} H_k{}^T \left(H_k P_{k/k-1} H_k{}^T + R_k \right)^{-1} \tag{S5}$$

$$\widehat{X}_k = \widehat{X}_{k/k-1} + K_k \left(Z_k - H_k \widehat{X}_{k/k-1} \right) \tag{S6}$$

$$P_k = \left(1 - K_k H_k\right) P_{k/k-1} \tag{S7}$$

Between the two control signals, the motion of the micro-robot can be regarded as a uniform acceleration motion, such that the state variable X of the system can be written as

$$X = [x, y, v_x, v_y, a_x, a_y] \tag{S8}$$

where x and y describe the position of the micro-robot, v_x and v_y describe the speed of the micro-robot, and ax and ay describe the acceleration of the micro-robot.

When the micro-robot is in uniform accelerated motion, the time-sampling interval is the control-time interval, $t = t_k + (1 - t)_k$, and its state equation is

$$\begin{cases} a_x = \ddot{x}, a_y = \ddot{y}, a_z = \ddot{z} \\ v_{x_{t+1}} = v_{x_t} + a_x t \\ v_{y_{t+1}} = v_{y_t} + a_y t \\ x_{t+1} = x_t + v_x t + \frac{1}{2} a_x t^2 \\ y_{t+1} = y_t + v_y t + \frac{1}{2} a_y t^2 \end{cases} \tag{S9}$$

Therefore, the state transition matrix Φ of the micro-robot is

$$\Phi = \begin{bmatrix} 1 & 0 & t & 0 & \frac{1}{2}t^2 & 0 \\ 0 & 1 & 0 & t & 0 & \frac{1}{2}t^2 \\ 0 & 0 & 1 & 0 & t & 0 \\ 0 & 0 & 0 & 1 & 0 & t \\ 0 & 0 & 0 & 0 & 1 & 0 \\ 0 & 0 & 0 & 0 & 0 & 1 \end{bmatrix} \tag{S10}$$

2. Simulation Results of the Rotating Magnetic Field

Figure S1 shows the simulation of the rotating magnetic field rotating about the z-axis. Since the period of one revolution is 1 s, we recorded the magnetic flux density distribution in the x–y plane every 0.125 s.

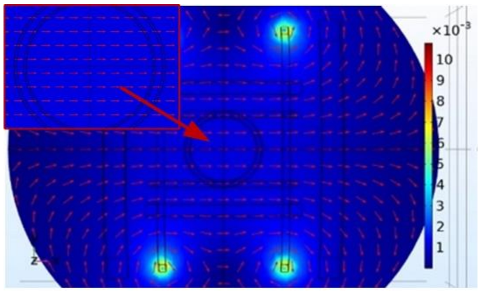

(a) t = 0.00 s

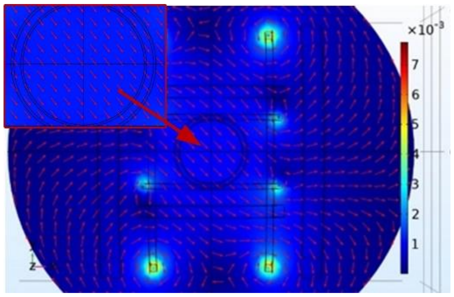

(b) t = 0.125 s

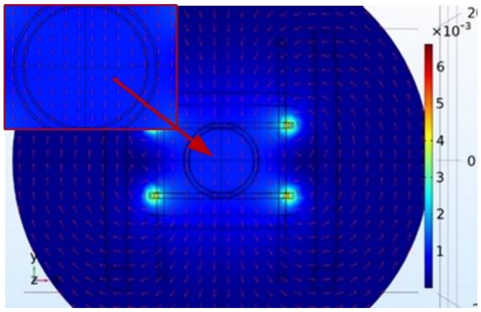

(c) t = 0.250 s

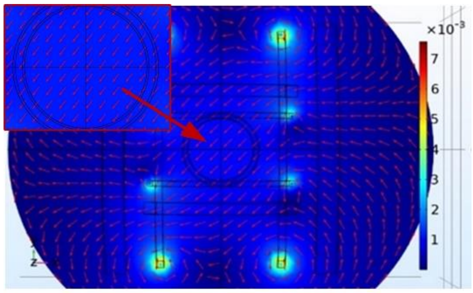

(d) t = 0.375 s

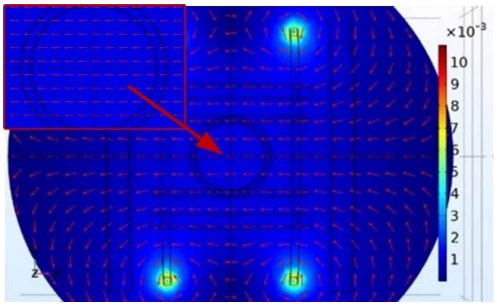

(e) t = 0.500 s

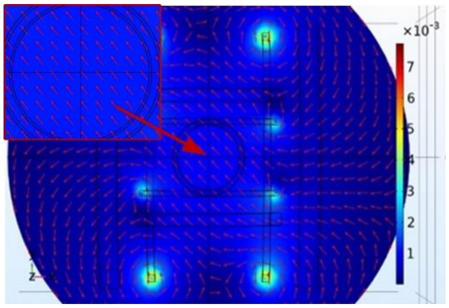

(f) t = 0.625 s

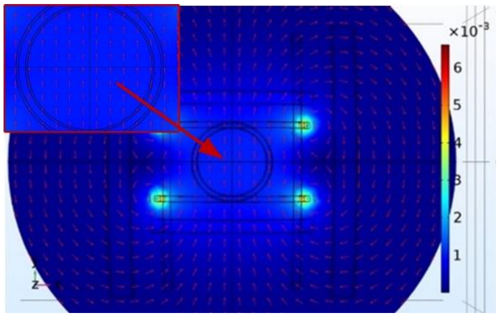

(g) t = 0.750 s

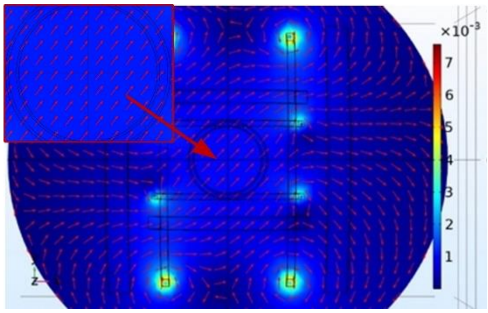

(h) t = 0.875 s

Figure S1. Rotating magnetic field.
